# Edible CaCO_3_ nanoparticles stabilized Pickering emulsion as calcium‐fortified formulation

**DOI:** 10.1186/s12951-021-00807-6

**Published:** 2021-03-04

**Authors:** Xiaoming Guo, Xiaoying Li, Leung Chan, Wei Huang, Tianfeng Chen

**Affiliations:** Department of Oncology, The First Affiliated Hospital, Department of Chemistry, Jinan University, Guangzhou, 510632 China

**Keywords:** Pickering emulsion, CaCO_3_, Gastrointestinal digestion, Bioresponsive

## Abstract

**Background:**

Nanoparticles assembled from food-grade calcium carbonate have attracted attention because of their biocompatibility, digestibility, particle and surface features (such as size, surface area, and partial wettability), and stimuli-responsiveness offered by their acid-labile nature.

**Results:**

Herein, a type of edible oil-in-water Pickering emulsion was structured by calcium carbonate nanoparticles (CaCO_3_ NPs; mean particle size: 80 nm) and medium-chain triglyceride (MCT) for delivery of lipophilic drugs and simultaneous oral supplementation of calcium. The microstructure of the as-made CaCO_3_ NPs stabilized Pickering emulsion can be controlled by varying the particle concentration (*c*) and oil volume fraction (*φ*). The emulsification stabilizing capability of the CaCO_3_ NPs also favored the formation of high internal phase emulsion at a high *φ* of 0.7–0.8 with excellent emulsion stability at room temperature and at 4 °C, thus protecting the encapsulated lipophilic bioactive, vitamin D3 (VD3), against degradation. Interestingly, the structured CaCO_3_ NP-based Pickering emulsion displayed acid-trigged demulsification because of the disintegration of the CaCO_3_ NPs into Ca^2+^ in a simulated gastric environment, followed by efficient lipolysis of the lipid in simulated intestinal fluid. With the encapsulation and delivery of the emulsion, VD3 exhibited satisfying bioavailability after simulated gastrointestinal digestion.

**Conclusions:**

Taken together, the rationally designed CaCO_3_ NP emulsion system holds potential as a calcium-fortified formulation for food, pharmaceutical and biomedical applications.
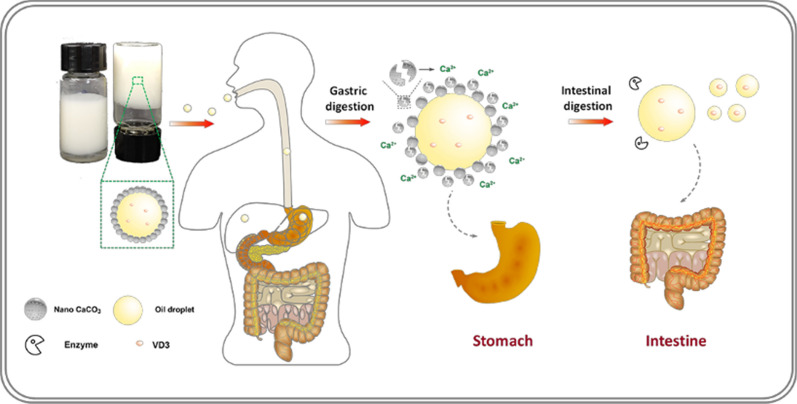

## Background

Pickering emulsions, a type of emulsion stabilized by solid particles rather than molecular surfactants, have received growing attention in the food, cosmetic, and biomedical industries due to their stability, which ranges from months to years, high internal payload capacity and added functionality derived from the stabilizing particles themselves [1–4]. In the field of drug oral delivery and release, Pickering emulsions enable the delivery of cargo to sites of interest (e.g., small intestine and colon) through passive diffusion-controlled or actuated release manners [1], relying on the combination of emulsifier properties and external triggers [5, 6]. When ingested, the stabilizing particles generally experience complex physicochemical changes as they are sequentially exposed to the human gastrointestinal (GI) tract, which encompasses the mouth, stomach, and small intestine [7–9]. GI-responsive fashion seems feasible if the particles can respond to these ‘internal’ stimuli from the GI environment. To achieve this goal, acid-sensitive Pickering emulsion system designs have been developed based on an environment that has many acid-labile particles. However, most of these particles were fabricated from synthetic materials that may raise safety concerns. Therefore, exploiting acid-sensitive Pickering emulsifiers that are non-toxic, biologically benign and compatible is of practical significance.

In the recent decade, there has been growing research interest in assembling particles using ‘‘generally regarded as safe’’ (GRAS) inorganic materials, such as hydroxyapatite, calcium phosphate, tri-calcium phosphate, and calcium carbonate, to maximize emulsion stabilization and minimize side effects [10–13]. Among the vast variety of materials, food-grade calcium carbonate (CaCO_3_) has gained attention as a Pickering emulsifier, particularly in the food, pharmacy, and medical industries [14], as it is of substantial importance for three main reasons: (1) CaCO_3_ is a recognized food additive and pharmaceutical ingredient that is advantageous for its low-cost and availability in large quantities [3]; (2) CaCO_3_ particles can impart acid-responsive properties into the Pickering emulsion it stabilizes by virtue of its acid-soluble nature [15]; (3) functional properties (calcium fortification and acid neutralization capacity) can also be added to the Pickering emulsion after converting CaCO_3_ particles into absorbable calcium ions once exposed to gastric acid [16]. Until now, CaCO_3_-based oil-in-water and water-in-oil Pickering emulsions have been reported [3, 17–19]. However, understanding the gastrointestinal fate of the CaCO_3_ Pickering emulsion has been minimally investigated. In-depth understanding of how the particle characteristics, such as the density, size, and surface properties, affect its gastric digestion behavior is necessary given that oral ingestion is the preferred route for CaCO_3_ Pickering emulsions.

Particle size control is crucial for the use of solid CaCO_3_ as a Pickering emulsifier [2, 18, 20, 21]. Generally, the particle size should be at least one order of magnitude smaller than the targeted emulsion droplet size [2]. Nanosized CaCO_3_ of different shapes (cube, sphere, and rod) has higher capabilities of forming a Pickering emulsion (mostly oil/water type) due to its finer size and partial wettability between both continuous and dispersed phases as compared to conventional CaCO_3_ of micron-scale features [18, 22] as it allows CaCO_3_ nanoparticles (NPs) to locate the droplet surface [20]. Another unique feature of CaCO_3_ NPs is its large surface area, which offers hydrophobization availability to enhance the emulsification efficacy [3, 17, 23]. From the viewpoint of calcium-fortified food, CaCO_3_ NPs would add value to the resulting Pickering emulsion as it favors a good calcium absorption rate, which has been supported by *in vivo* animal and clinic studies [24, 25].

In this study, we have reported a food-grade Pickering emulsion using CaCO_3_ NPs as the stabilizer. First, we synthesized the CaCO_3_ NPs (40–140 nm) through the in situ reaction of CO_2_ and calcium hydroxide in an aqueous solution. Next, the CaCO_3_ NPs were successfully used to prepare Pickering emulsions at oil volume fractions ranging from 0.2 to 0.8. Then, the CaCO_3_ NP-based Pickering emulsion was used as a vehicle for delivering vitamin D3 (VD3) through the GI tract. VD3 was specially selected as a model compound, as it is of importance for two reasons: on the one hand, VD3 is well-soluble in oil and thereby is advantageous for the assessment of its bioavailability in the in vitro simulated GI analysis [26–28]; on the other hand, as VD3 is frequently formulated with CaCO_3_ as a co-medication [29, 30], it could shed light on the conceptualization of the Pickering emulsion with enhanced calcium fortifying properties based on CaCO_3_ NPs and VD3. The potential of CaCO_3_ NP-based stabilized Pickering emulsion as an acid-responsive delivery system for food, and medical applications was also discussed.

## Materials and methods

### Materials

Food-grade calcium hydroxide slurry (8 % w/w) was supplied by Enping Hui Xiang lndustrial Co., Ltd. (Jiangmen, China). Medium-chain triglyceride (MCT; C8:C10 = 60:40) with a density of 0.943 g·mL^− 1^ was purchased from Britz Networks Sdn. Bhd (Melaka, Malaysia) and used as received. Food additive grade commercial CaCO_3_ powder (> 99 %), referred to as CCaCO_3_, was purchased from Jiarun Biotechnology (Henan, China). Tween 80 (TW80) was obtained from Aladdin (China, T104865). VD3 (cholecalciferol) (> 98 %) was obtained from Macklin (China, C804669). Enzymes and reagents used in simulated gastric and intestinal digestions were hydrochloric acid (Guangzhou Chemical Reagent Factory, China), pepsin (> 400 U/mg; Sigma-Aldrich, USA, P7000), bile salt (Sigma-Aldrich, USA, 48,305), lipase (30–90 U/mg; Sigma-Aldrich, USA, L3126). Other reagents used included curmarin-6 (Cur-6; Aladdin, China, C100929), rhodamine B (RB; Aladdin, China, R104960), phosphate-buffer saline (PBS; Beyotime, China, C0221A), methanol (Merck, Germany, 106,007), methanesulfonic acid (Thermo, USA, 057558), and Nile red (Sigma-Aldrich, USA, 72,485). Ultrapure water was used in all experiments.

### Pilot-scale preparation of CaCO_3_ NPs

**Fig. 1:**
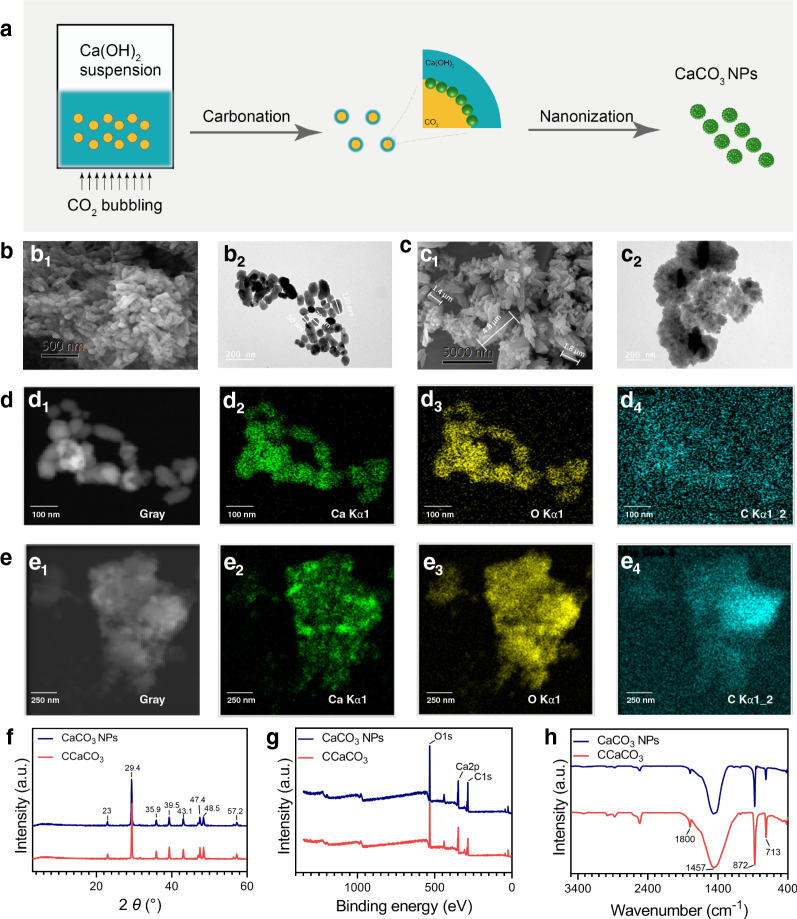
Fabrication and structure/composition characterization of the CaCO_3_ NPs and CCaCO_3_. **a** Schematic illustration of the fabrication process of the CaCO_3_ NPs. SEM (**b**_**1**_ and **c**_**1**_) and TEM (**b**_**2**_ and **c**_**2**_) images of CaCO_3_ NPs and CCaCO_3_, respectively. TEM/EDS elemental mapping images of the **d** CaCO_3_ NPs and **e** CCaCO_3_. **f** XRD, **g** XPS, and **h** FTIR spectra of the CaCO_3_ NPs and CCaCO_3_

CaCO_3_ NPs were synthesized by the gas-solid-liquid carbonation method (Fig. 1a) using a pilot-scale setup. CO_2_ (15 L/min) was bubbled into 120 L of calcium hydroxide at an appointed concentration of 4 % (w/v) in the crystallizer to start the carbonation reaction under a controlled temperature of approximately 25 ± 2°C maintained by circulating water. The carbonation was kept for ~ 10 min until complete reaction was achieved, as verified by the decrease in system pH to approximately 7. For particle aging, the slurry was transferred to an open tank and stirred at 250 rpm for 12 h until the pH was stable at 7. Afterward, the suspension was centrifuged at 5,000 *g* for 20 min, vacuum-dried for 12 h, and ground to pass through a 200-mesh screen. The pulverized powder was stored until further use.

### Structural and morphologic characterization

The crystalline structure of CCaCO_3_ and CaCO_3_ NPs was measured using an Ultima IV X-ray diffractometer (XRD, Raguku, Japan). X-ray photoelectron spectroscopy (XPS) measurements were carried out using a K-alpha^+^ spectrometer (Thermo Scientific, USA). Fourier transform infrared spectra (FTIR) of the samples were measured using a Tensor 27 spectrometer (Bruker, Germany). Nanostructures of CaCO_3_ NPs and CCaCO_3_ were captured by a TECNAI G2 F20 transmission electron microscopy (TEM) (Phenom, the Netherland). Samples were suspended into aqueous solutions and loaded onto a 200-mesh copper grid. Energy-dispersive X-ray spectroscopy (EDS)-mapping analyses were performed at several locations on the substrate to confirm the presence of calcium, oxygen, and carbon. Scanning electron microscopy (SEM) images were taken with a field emission scanning electron microscopy (LE, Phenom, the Netherland). The samples were carefully mounted on a copper grid, which was stuck on double-sided carbon tape and sputter-coated with gold before scanning. The mean particle size of CaCO_3_ NPs was measured and calculated using the Nano Measurer software (Version 1.2).

### Preparation of the CaCO_3_ NP-based stabilized emulsions

Preliminary experiments on the structural stability of the CaCO_3_ NPs were conducted in aqueous solutions at pH = 7.4, 6.8, and 5.3. According to TEM data (Additional file 1: Figure S1), surface erosion was evident for the CaCO_3_ NPs at pH 5.3. This finding predicts CaCO_3_ NP bulk disintegration at pH levels below 5.3 [11]. Since structural intactness is the basic prerequisite for a particle Pickering emulsifier, acidic pH conditions were not taken into account, and the emulsification performance of the CaCO_3_ NPs was investigated at pH 7.4. Conveniently, weighted CaCO_3_ NP powder was stirred into PBS (20 mmol·L^− 1^, pH 7.4) at a concentration (*c*) of 1–4 % (w/v) and then passed through a UH-5 homogenizer (Union-Biotech Co., Ltd, Shanghai, China) equipped with a diamond-coated valve to disrupt the formed micron aggregates. The homogenization was conducted at 120 MPa for three passes. Sodium azide (0.02 % w/v) was added as the preservative. The aqueous phase prepared above was mixed with MCT (to yield a total volume of 20 mL) at an oil volume fraction (*φ* = V_oil_ / (V_oil_ +V_water_)) between 0.2 and 0.9, with an increment of 0.1. The two-phase mixture was homogenized by a handheld dispenser (Heidolph, SilentCrusher M, Germany) at 8,000 rpm for 2 min. The shearing condition was selected based on the trends of mean droplet size and droplet size distribution (DSD) with increasing shearing speed from 5,000 to 20,000 rpm (Additional file 1: Figure S2). In all emulsions, the *c* refers to the continuous phase rather than the whole emulsion. Emulsions were transferred to plastic tubes or screw-cape glass vials for measurements of mean droplet size ("Emulsion characterization" section), visual appearance, and microscopic observation ("Emulsion characterization" section) at set time intervals (0 and 30 days). Selected emulsions were stored at room temperature (RT) and 4 °C for the characterization of emulsion stability. With CCaCO_3_ or TW80 as emulsifiers, two control groups were set for comparison (Additional file 1: Figures S3 and S4). For these two control groups, shearing conditions (speed and time) were the same as those of the CaCO_3_ NPs group.

### Emulsion characterization

The DSD, Sauter mean diameter (d_32_) [31], and specific surface area of the emulsion were measured using a Mastersizer 3000 particle sizer (Malvern, UK) with the refractive indexes of 1.33 and 1.45 for water and MCT, respectively. Bright field microscopic observations were carried out using an EVOS FL microscopy (ThermoFisher, USA). An aliquot of emulsion (5 µL) was carefully taken from each examined sample, pitted onto a glass slide, covered with a cover glass, and imaged under bright field mode. To visually evaluate the stability of emulsion often perceived as creaming, sediment, and demulsification into an oil layer, the emulsions were placed in plastic centrifugal tubes, and then their visual appearance was imaged by a digital camera. Storage and loss moduli of emulsions were measured using an AR1500EX rheometer (TA Instruments, USA) equipped with a parallel plate geometry (diameter: 40 mm, gap: 1 mm). A 1 mL emulsion was loaded onto the plate and covered with silicone oil around the edges of the sample to avoid solvent evaporation. All measurements were conducted at 25 °C. A set of emulsions (*φ* = 0.8) stabilized by TW80 at concentrations of 0.125–1 % w/v served as control.

### Centrifugation stability

Centrifugation stability measurement was performed on two representative emulsions stabilized by either CaCO_3_ NPs or TW80 (control). Briefly, each tested emulsion (5 mL) was stored in a 15-mL screw-capped centrifuge tube and then centrifuged at 4000 *g* [32, 33] for 5 min. The centrifugal instability of emulsion was assessed based on the creaming index, which is calculated by the following equation [32].

$${\%} \text{Creaming}\,\,\text{index}= \frac{{H}_{s}}{{H}_{t}}\times 100,$$

where *H*_*s*_ and *H*_*t*_ represent the height of the lower aqueous phase layer and the total height of the centrifuged emulsion.

### Stability of emulsion upon dilution

Emulsion stability upon dilution and storage was studied for the CaCO_3_ NPs stabilized emulsion (*φ* = 0.8, *c* = 4 % w/v) and for the TW80 control (*φ* = 0.8, *c* = 0.5 % w/v). Each emulsion type was freshly prepared and then separately diluted 2, 3, and 5 times with ultrapure water and stored at RT for 7 days. Mean droplet diameter and DSD were measured for the two parent emulsions and their diluted emulsions before and after storage.

### Preparation and characterization of VD3-encapsulated emulsion

The VD3 loaded Pickering emulsion was formulated with 14 g MCT oil containing 40 mg VD3 as the oil phase and 5.96 g of CaCO_3_ NP suspension (*c* = 4 % w/v) to yield a total weight of 20 g and a VD3 concentration of 2.0 mg/g of the whole emulsion. A control group was specifically prepared in the same manner as that of CaCO_3_ NPs except using TW80 (*c* = 0.5 % w/v) solution instead of CaCO_3_ NPs dispersion as the continuous phase. A 2 mg/mL VD3 containing MCT oil was also used as a control group. Each sample to be tested was immediately transferred into screwed glass vials for avoiding excessive exposure to oxygen and then stored at 4 °C and RT. At the pre-designated time points, an aliquot (1 g) of the stored emulsion was withdrawn and then mixed with ethanol (1:9 by vol) with assisted vortexing (30 s) to extract VD3 and MCT from the emulsion. After centrifugation at 15,000 *g* for 15 min, the supernatant was diluted 10-fold with ethanol, passed through a 0.22 µm filter, injected into a high-performance liquid chromatography (HPLC) system equipped with an Atlantic T3-C18 column (4.6 × 250 mm, 5 µm, Waters, USA), and isocratically eluted by methanol at a flow rate of 1.0 mL/min. The quantification of VD3 was achieved using an ultraviolet (UV) detector (Agilent Technologies, USA) at 265 nm (R^2^ = 0.9999). The content of the remaining VD3 was calculated accordingly to the following equation:

$${\%} \,\text{remaining}\, \text{VD}3=\frac{{W}_{i}}{{W}_{0}}\times 100,$$

where *W*_*0*_ and *W*_*i*_ represent the total feeding weight of VD3 before storage and the remaining weight of VD3 after storage, respectively.

### Simulated GI digestion

The digesting behaviors of VD3-loaded CaCO_3_ NP-based stabilized emulsion (*φ* = 0.8, *c* = 4 % w/v) were studied using the two-stage *in vitro* digestion model based on previous research [34, 35] with slight modifications. A VD3-loaded TW80-stabilized emulsion (*φ* = 0.8, *c* = 0.5 % w/v) and a VD3 powder were used as controls. Assessment of the visual appearance and quantification of the VD3 content were performed at the end of the following two processes.

Gastric digestion. Briefly, the emulsion was diluted 40-fold with ultrapure water and then incubated in a water bath at 37 °C. Ten mL of the diluted and prewarmed emulsion (containing 0.18 g oil) was mixed with 10 mL simulated gastric fluid (pH 1.2; 34 mmol·L^− 1^ NaCl, and 3.2 mg·mL^− 1^ pepsin) [36] in a 100 mL glass beaker; this mixture was adjusted to pH 2.5 using NaOH solutions and then incubated at 37 °C with stirring (1,000 rpm) for 1 h. At the end of digestion, a 250 µL aliquot sample was withdrawn from the aqueous phase for calcium quantification by the established HPIC procedure (Additional file 1: Figure S5), to which an equal volume of phosphate buffer was supplemented to the digestive fluid.

Intestinal digestion. The whole gastric digested mixture was adjusted to pH 7.0 and mixed with 1.5 mL of simulated intestinal fluid (containing 250 mM CaCl_2_ and 3 M NaCl), and supplemented with 3.5 mL of bile salts (54 mg·mL^− 1^) [34]. After pH readjustment to 7.0, 2.5 mL of the lipase solution was added to start the lipolytic reaction at 37 °C for 2 h with constant stirring at 250 rpm. An autotitrator (Metrohm Ltd., USA) was used to monitor the pH during the whole digestion period and adjusted the pH back to 7.0 by titration with a 0.1 M NaOH solution. The amount of free fatty acids (FFA) produced by lipolysis based on its added volume into the reaction vessel was calculated by the following equation [35]:

$${\%} \text{FFA}=\frac{({C}_{NaOH}\times {V}_{NaOH}\times {Mw}_{Lipid})}{({W}_{Lipid}\times 2)}\times 100,$$

where C_*NaOH*_ is the molarity of the NaOH solution employed (0.1 M), *V*_*NaOH*_ (L) is the volume of NaOH required to neutralize FFA released, *Mw*_*Lipid*_ is the average molecular weight of MCT (503.9 g·mol^− 1^) calculated based on the C8: C10 proportion of MCT, and *W*_*Lipid*_ is the total weight of MCT (0.18 g) used for digestion.

### Fluorescent microscopy

The microstructure of the emulsions after in vitro gastric and intestinal digestions was measured using fluorescent microscopy (EVOS FL, ThermoFisher, USA). All ingredients and their concentrations were the same as in "Simulated GI digestion" section except that the oily phase was stained with Nile red before emulsification. The dyed emulsion was treated in the same manner as "Simulated GI digestion" section. At each phase, the visual appearance of the digested emulsion was analyzed, and the emulsion microstructure was analyzed under red fluorescence imaging. To evidence the adsorption of CaCO_3_ NPs onto oil droplets, we examined two selected emulsions (*φ* = 0.8) stabilized by CaCO_3_ NPs (*c* = 4 % w/v) and TW80 (*c* = 0.5 % w/v), respectively, using a confocal laser scanning microscope (LSM 880 with AiryScan, Carl Zeiss). Before emulsification, the oil phase of both emulsions was dyed by Cur-6, while RB dyed the continuous phase containing CaCO_3_ NPs. Fluorescence from Cur-6 and RB was excited at 458 nm and 543 nm, respectively.

### Animal study and hematology analysis

The in vivo oral toxicity of CaCO_3_ NPs stabilized emulsion was evaluated using a mouse model with the approval of the Laboratory Animal Ethics Committee of Jinan University. Four-week-old female ICR mice, purchased from GemPharmatech Co., Ltd. (Jiangsu, China), were randomly divided into four groups. Mice of the three treated groups were gavaged with the CaCO_3_ NPs stabilized emulsion (*φ* = 0.8 and *c* = 4 % w/v) at daily doses of 325, 650, 1300 mg/kg (CaCO_3_ dose), respectively, which equates to 1, 2, and 4 times of the daily calcium dose for adult as recommended by the National Institutes of Health (NIH). The mice of the control group received 0.9 % NaCl per day. After 7-day gavage treatment, the mice were euthanized; blood samples were collected, centrifuged at 3000 rpm × 15 min at 4 °C to obtain serum, and sent to Servicebio Biotechnology Co., Ltd (Wuhan, China) for hematological analysis. Besides, major organs were isolated, fixed in 4% paraformaldehyde, and stained with hematoxylin and eosin (H&E).

### Statistical analysis

Unless otherwise stated, all data presented are the mean of three independent triplicates. Statistical evaluation was performed using the SPSS Statistics software (version 20.0, IBM Corp., USA). The difference between means was analyzed by a one-way analysis of variance (ANOVA)/Duncan’s test (*p* < 0.05).

## Results and discussion

### Preparation and characterization of the CaCO_3_ NPs

The as-prepared CaCO_3_ NPs had an ellipsoidal geometry with sizes ranging from 40 to 140 nm, as analyzed by the SEM and TEM analyses (Fig. 1b). Similar morphologies have been reported for the CaCO_3_ NPs [37]. In contrast, CCaCO_3_ displayed amorphous structures of ununiform sizes, of which some had diameters greater than 1 µm (Fig. 1c). The three constituent elements, namely Ca, O, and C, were distributed in both the CaCO_3_ NPs and CCaCO_3_ as analyzed by the EDS elemental mapping (Fig. 1d and e). Nevertheless, the C signal was negligible for the CaCO_3_ NPs due to the background noise generated from the carbon supporting film of the copper grid. Quantification of the calcium content showed that calcium represented 39.4 ± 0.9 % and 38.3 ± 1.2 % of the CaCO_3_ NPs and CCaCO_3_ (Additional file 1: Figure S5), respectively, which agreed well with that of pure anhydrous calcium carbonate. The CaCO_3_ NPs and CCaCO_3_ exhibited similar X-ray diffraction patterns consisting of characteristic peaks at 29.4°, 35.9°, 43.1°, 47.4°, 48.5°, and 57.2° (Fig. 1f), which corresponded to calcite [38]. The XPS (Fig. 1g) and FTIR (Fig. 1h) analyses indicated structural similarity between the CaCO_3_ NPs and CCaCO_3_, in which the CaCO_3_ NPs and CCaCO_3_ exhibited similar spectroscopic data. All of the results demonstrated the successful fabrication of nanoparticulate CaCO_3_ with almost identical compositional and crystalline properties but different morphologies.

### Preparation of the CaCO_3_ NP-based stabilized emulsions

Although various oils, including soy, corn, olive oils, MCT, etc. can be solvents for VD3, it should be mentioned that MCT is more applicable to carry VD3 due to its oxidation stability [39]. As a result, any adverse effect of lipid oxidation on the stability of VD3 can be ruled out. Also, as an oil phase, MCT is readily soluble in ethanol (Additional file 1: Figure S6); thus, it offers the convenience of injecting the VD3 containing MCT-ethanol solution onto the C18 analytical column without solvent evaporation ("Preparation and characterization of VD3-encapsulated emulsion" section). For these reasons, MCT was selected as the preferred oily phase in this study.

**Fig. 2:**
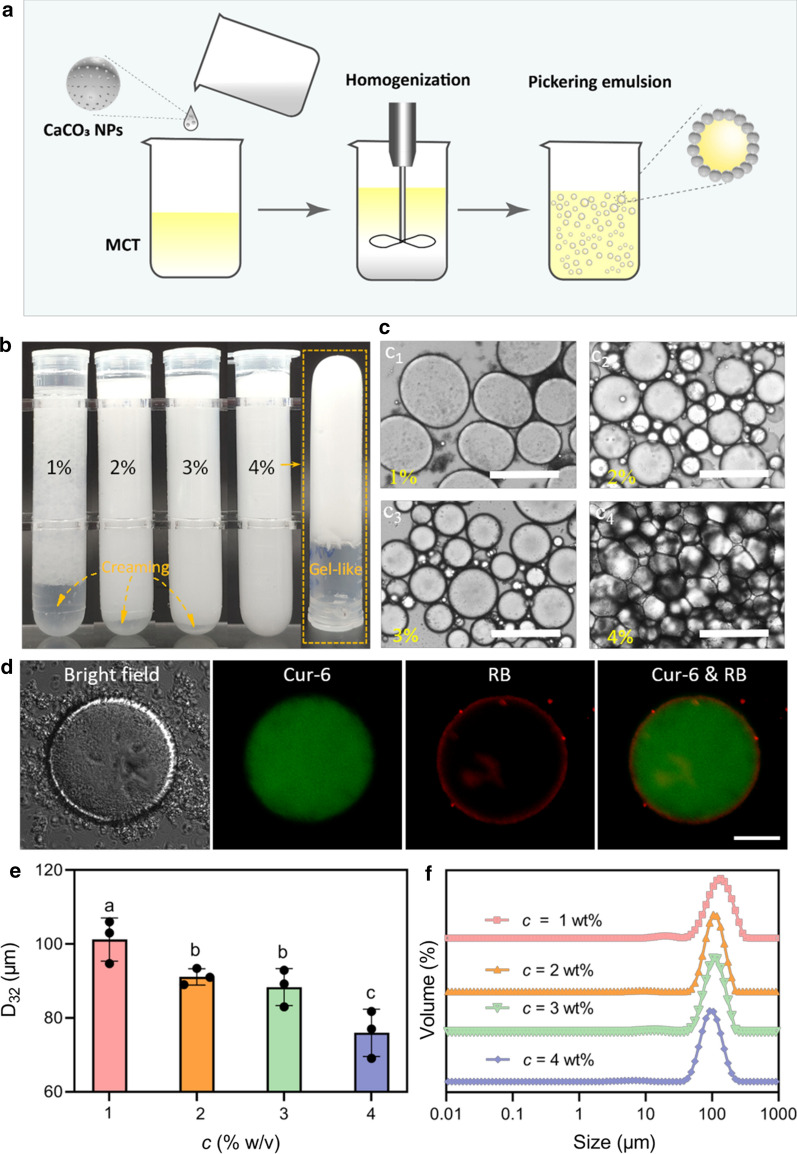
Preparation and characterization of the Pickering emulsions stabilized by the CaCO_3_ NPs. **a** Schematic illustration of the fabrication process of the CaCO_3_ NP-based stabilized Pickering emulsion. **b** Visual appearance of creamed and homogenous emulsions (*c* = 1–4 % w/v and *φ* = 0.8). The right panel shows the gel-like emulsion prepared with the highest *c* (4 % w/v). **c** Micrographic images showing the microstructure of emulsions, as presented in **b**. Scale bars are 200 µm. **d** Confocal laser scanning microscope images of droplets stabilized by CaCO_3_ NPs. The oil droplet dyed by Cur-6 showed green fluorescence, whereas the CaCO_3_ NPs dyed by RB showed red fluorescence. The scale bar is 30 µm. **e**, **f** Mean droplet diameter (d_32_) and DSD of CaCO_3_ NPs stabilized emulsions as a function of *c*. The particle concentration refers to the particles or surfactant concentration in the continuous phase rather than the whole emulsion. Data are mean ± standard deviation in triplicate. Bars with different letters (a, b, and c) indicate statistically significant (*p* < 0.05) differences among samples

Previous studies [3, 17–20] have indicated that the CaCO_3_ NPs render the formation of O/W emulsion. Therefore, we evaluated the ability of the CaCO_3_ NPs and CCaCO_3_ to emulsify the system with a *φ* of 0.8. The two phases were homogenized upon mechanic shearing (schematically shown in Fig. 2a) in the presence of the CaCO_3_ NPs rather than CCaCO_3_. Hence, CCaCO_3_ was incapable of forming an emulsion even after increasing the particle concentration to 4 % w/v (Additional file 1: Figure S3a), where most of the CCaCO_3_ remained in the lower aqueous phase, as evidenced under microscopy observation (Additional file 1: Figure S3b). Instead, the CaCO_3_ NPs resulted in emulsion formation at *c* = 1–4 % (Fig. 2b), and all of the emulsions (except that prepared with *c* = 4 % w/v) creamed right after preparation for storage of 12 h at room temperature. For the three creamed emulsions, no CaCO_3_ NPs were detectable at the bottom of their aqueous lower layers, indicating that CaCO_3_ NPs stayed in the emulsified phase, probably adsorbing on oily surfaces. Notably, the emulsion with *c* = 4 % was gel-like, displaying self-supporting properties. To study the interfacial behaviors of CaCO_3_ NPs at different concentrations, we measured the interfacial tensions between MCT and different CaCO_3_ NPs dispersions (1–4 % w/v; see Additional file 1: Figure S7). The results showed that the interfacial tension values decreased upon CaCO_3_ NPs addition in a concentration-dependent manner. To gain insight into such a unique texture of the ‘gelled’ emulsion, we visualized the emulsions under optical microscopy. The microscopic results (Fig. 2c) showed that oil droplets of different sizes were well distributed in all of the emulsions, but those contained in the concentrated (*c* = 4 % w/v) emulsion were stuck to each other very firmly, forming a gel-like network that restricted oil droplet development (Fig. 2c_4_) [40]. Through this densely packed manner, the oil droplets exhibited difficulty in moving, thereby maintaining the hardly flowing emulsion. According to previous findings, the critical *φ* required to form a high internal phase emulsion (HIPE) was around 0.74 (maximum of 0.80), where a gel-like network was formed due to the solid concentration-related oil droplet flocculation [40–42]. The red fluorescence of the image identified the RB-dyed CaCO_3_ NPs, and the overlapped fluorescence of RB (red fluorescence) and Cur-6 (green fluorescence) evidently showed the adsorption of CaCO_3_ particles at the boundary of oil droplet (Fig. 2d). Decreasing d_32_ values, which ranged from 76 µm to 101.2 µm, were detected at increasing *c* for the emulsions (Fig. 2e). The four emulsions had similar monomodal droplet size distributions, though the peak positions slightly shifted to smaller sizes at higher *c* values (Fig. 2f). At a lower *c* of 0.5 % w/v, CCaCO_3_ still did not form an emulsion, whereas CaCO_3_ NPs only generated a thin emulsified layer located between the upper oily and lower aqueous phases (Additional file 1: Figure S3c).

**Fig. 3:**
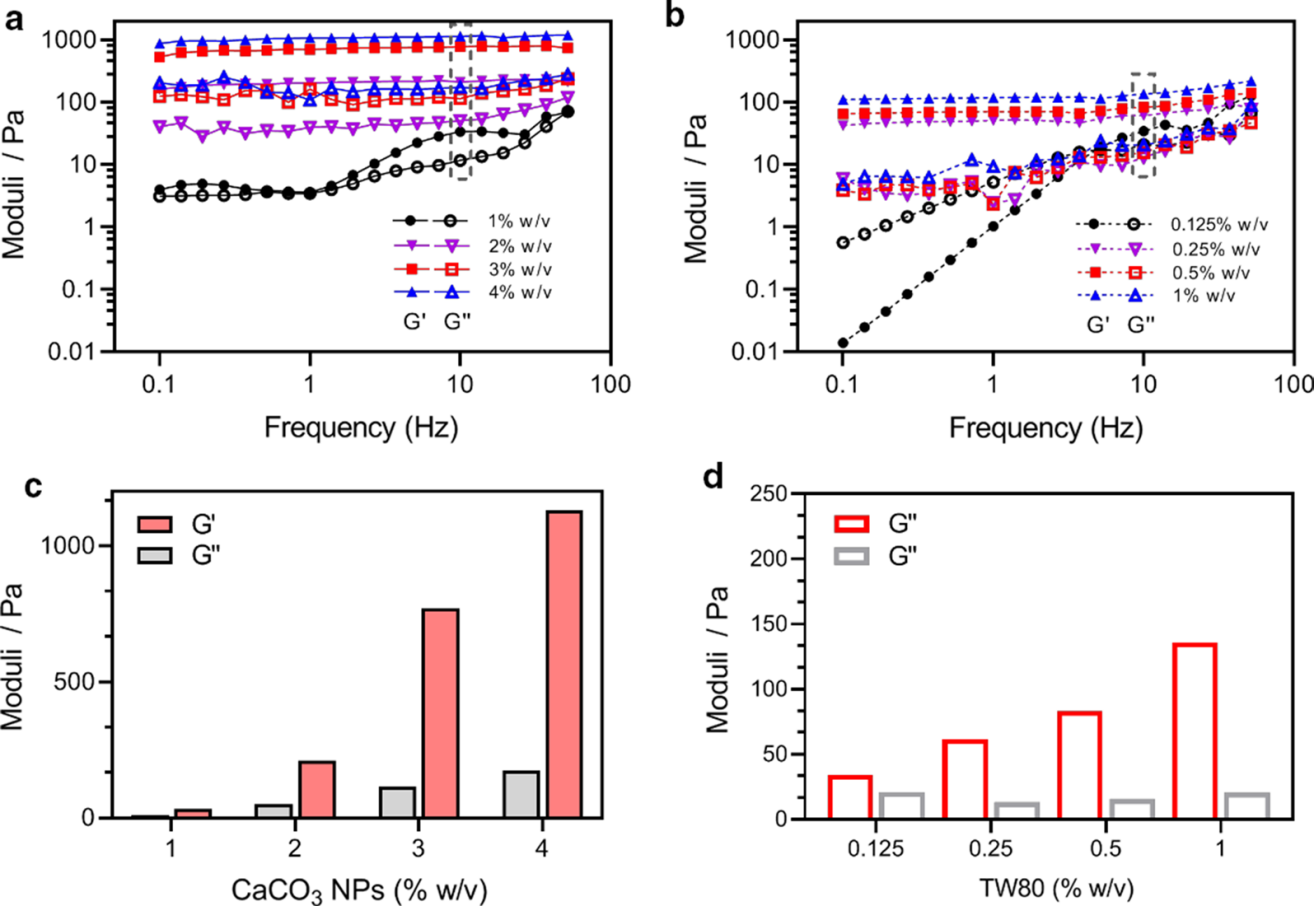
Comparison of the viscoelastic properties between HIPEs prepared with CaCO_3_ NPs and TW80. **a**,** b** Dependence of storage moduli (G’) and loss moduli (G’’) on shear frequency for the two serious of HIPEs (*φ* = 0.8). **c**,** d** Values of G’ and G’’ recorded at 10 Hz for the same emulsions in **a** and **b**. The data were measured at a fixed shear strain of 0.5 %

Figure 3 shows the dependence of G’ and G’’ on the shearing frequency for the emulsions prepared at different *c* values. G’ was always higher than G’’, irrespective of the *c* used, and both G’ and G’’ were proportional to *c* (Fig. 3a). Interestingly, the largest increment in either G’ or G’’ occurred at *c* = 3 % (Fig. 3c). Since the d_32_ values at concentrations of 2 % and 3 % w/v are similar, it should be the continuous phases affecting the viscoelasticity properties of emulsions. To support our supposition, we measured apparent viscosities for the four dispersions of CaCO_3_ NPs. The results showed that the viscosity increased rapidly when *c* increased from 2 to 3 % w/v (Additional file 1: Figure S8), probably due to particle aggregation. Therefore, based on these findings, we ascribe the sharp G’ or G’’ increments to the continuous phase’s thickening. For TW80 emulsions in the control group, the G’ also increased with increasing concentrations (Fig. 3b), but in a lower range. The G’ measured with TW80 at 1 % w/v was about one-tenth of that with 4 % w/v CaCO_3_ NPs (Fig. 3d). Emulsion viscosity/viscoelasticity has been well-demonstrated to be one of the affecting factors to emulsion stability against droplet coalescence [43, 44]. Based on these observations, the highest viscoelasticity with *c* = 4 % may result in the most optimal stability. The *c* was set 4 % w/v in the following experiments based on the creaming and viscoelasticity data.

**Fig. 4:**
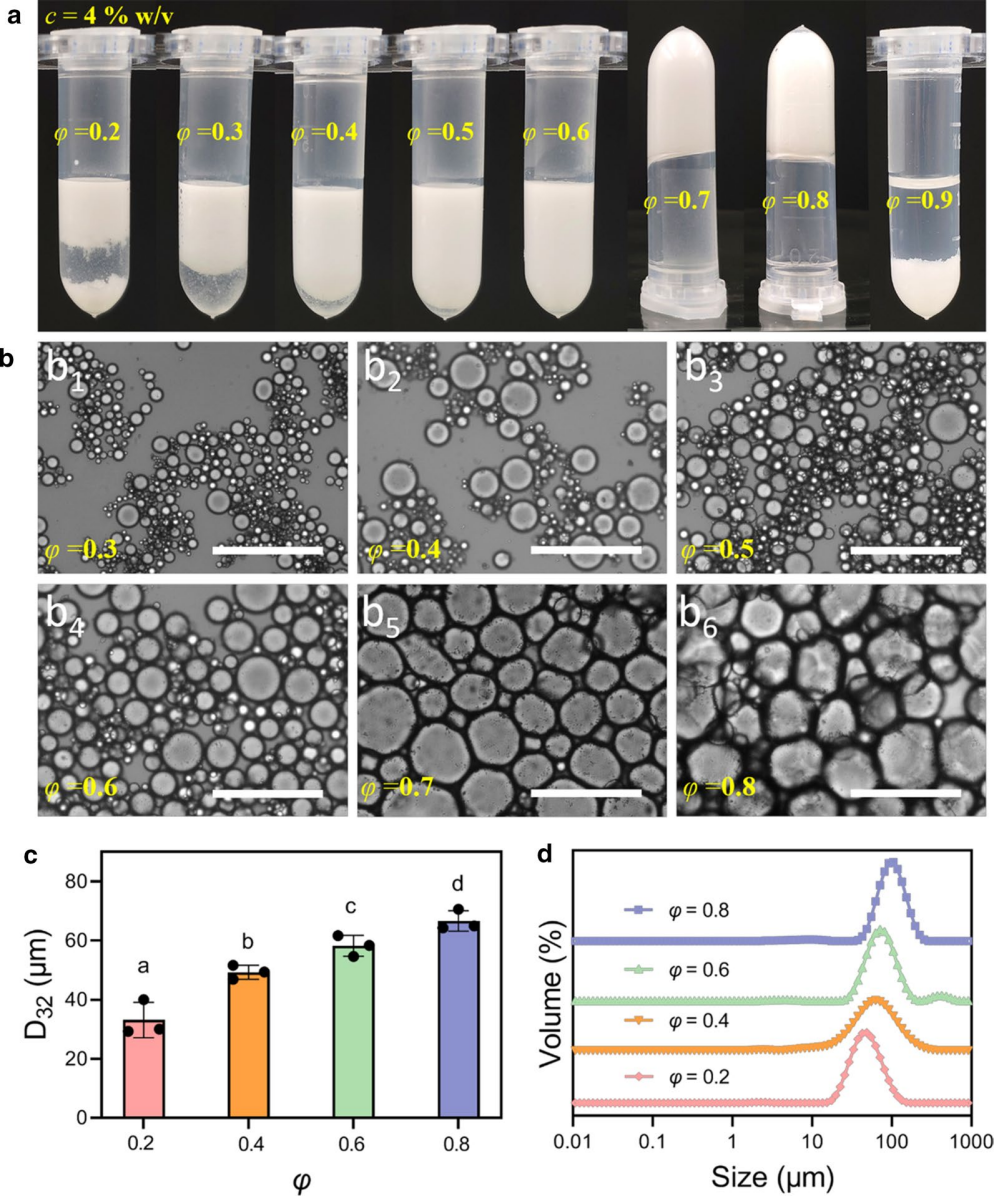
CaCO_3_ NP-based stabilized Pickering emulsion displayed ***φ***-dependent emulsification properties. **a** Visual appearance of emulsions (*c* = 4 % w/v) with *φ* ranging from 0.2 to 0.9. Samples were placed into vials just after preparation and then photos were taken after standing at room temperature for 12 h. **b** Micrographic images of the six representative emulsions (*c* = 4 % w/v and *φ* = 0.3–0.8). Scale bars: 200 µm. **c**, **d** d_32_ and DSD of the selected emulsions with *φ* increment of 0.2, between 0.2 and 0.8. Data are mean ± standard deviation of the triplicates. Bars with different letters (a, b, c, and d) indicate statistically significant (*p* < 0.05) different samples

The performance of the CaCO_3_ NPs as an emulsion stabilizing agent was observed within a wide range of *φ* = 0.2 to 0.9. The emulsions prepared at *φ* values lower than 0.5 creamed soon after preparation, and the creaming heights were proportional to *φ* (Fig. 4a). At *φ* = 0.2, parts of the upper creamed layer deposited after 12 h of standing at RT (Fig. 4a). Increasing *φ* to a higher range of 0.7–0.8 resulted in stable emulsions based on the absence of creaming even after storage for 30 days (data not shown). Notably, self-standing properties were imparted into the emulsions prepared at *φ* = 0.7–0.8. Further increasing *φ* up to 0.9, however, resulted in the presence of an emulsified layer as the lower phase, while most of the oily phase remained unemulsified. Microscopic data showed that the oil droplets were densely packed within the two HIPEs (*φ* = 0.7–0.8), whereas relatively small-sized droplets were present in low *φ* emulsions (*φ* = 0.3–0.6) (Fig. 4b). Analysis of the droplet characteristics further showed that the d_32_ values increased with increasing *φ* (Fig. 4c). The DSD patterns were nearly monomodal across a rather wide size ranging from 10s to 100s of µm (Fig. 4d). Based on the results of Fig. 4, it was concluded that emulsions of different droplet characteristics, specifically the droplet size and distribution could be fabricated by simply varying *φ*.

### Centrifugation and dilution stability

**Fig. 5:**
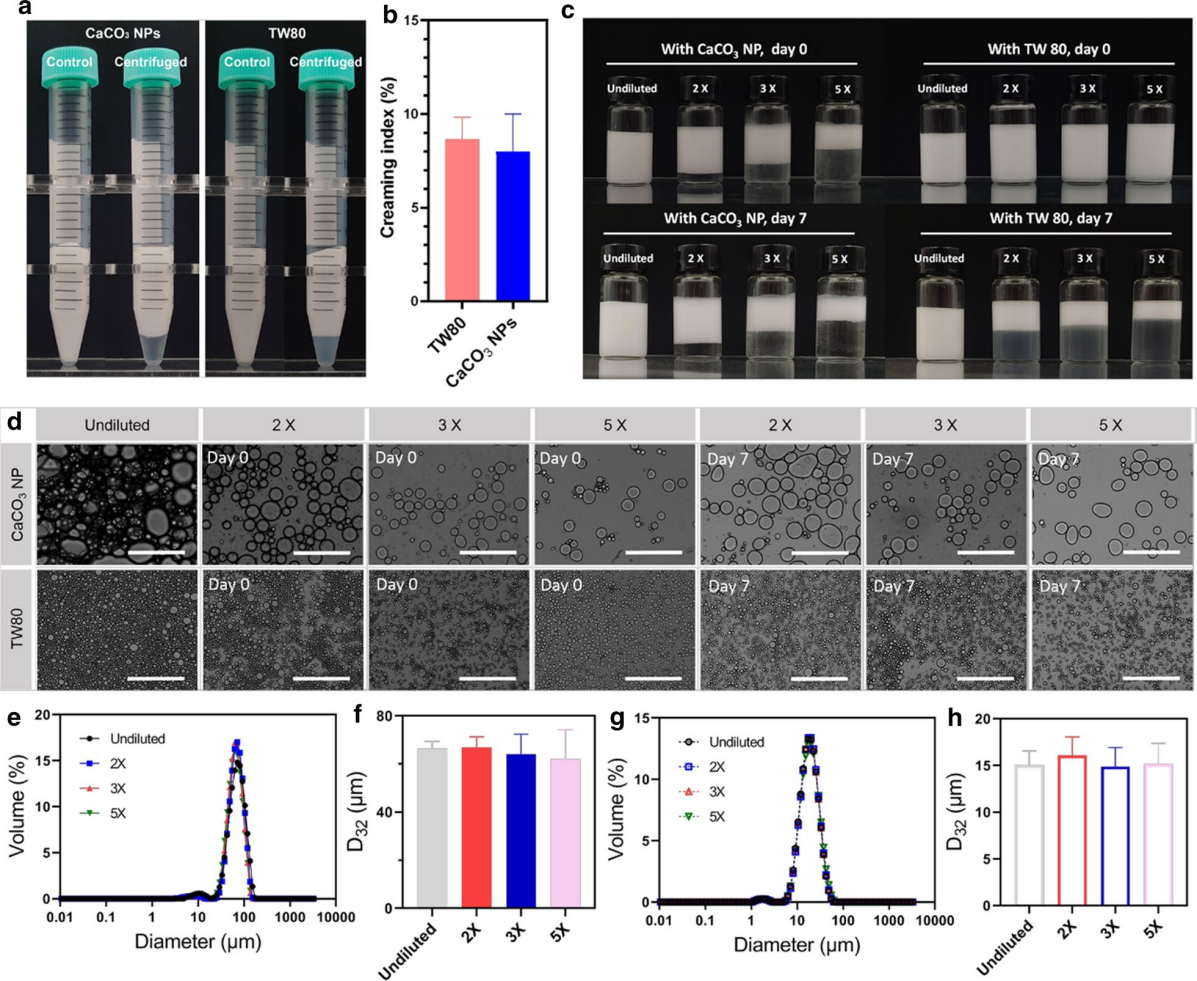
Stability against centrifugation and dilution of the emulsions stabilized by CaCO_3_ NP (4 % w/v) and TW80 (0.5 % w/v). **a** visual appearance of the two emulsions before and after centrifugation at 4000 *g* for 5 min, **b** creaming indices. Data are mean ± standard deviation of the triplicates. **c** Photographs of high internal phase and diluted MCT-water emulsions (*φ* = 0.8) stabilized by 4 % w/v CaCO_3_ NPs and 0.5 % w/v TW80 taken at day 0 and day 7 after preparation. **d** Micrographs of two selected emulsions (*φ* = 0.8) stabilized by CaCO_3_ NP and TW80, respectively, with different dilution ratios (2, 3, and 5 times) on day 0 and day 7 after preparation. Scale bars are 400 µm. Droplet characteristics of emulsions stabilized by CaCO_3_ NP and TW80, from **e** to **h**: DSD with CaCO_3_ NPs, mean droplet diameters with CaCO_3_ NPs, DSD with TW80, mean droplet diameters with TW80

Creaming is one of the emulsion-breaking mechanisms. In this study, both creamed and uncreamed emulsions were prepared, depending on the *φ* used (Fig. 4). To further analyze the creaming stability, we centrifuged the uncreamed one (*φ* = 0.8, *c* = 4 % w/v) at 4000 *g* for 5 min. For comparison, a 0.5 % w/v TW80 stabilized emulsion was prepared at the same *φ* and treated in the same manner. As shown in Fig. 5a, each of the initial uncreamed emulsions separated into two phases after centrifugation, but no sedimentation occurred. No significant difference in creaming index was observed for the two examined emulsions (Fig. 5b).

Concentrated emulsions usually need to be diluted into thin forms before use. Emulsion dilution, nevertheless, may destabilize the stability of the droplets. Hence, it is of significance to investigate the dilution stability of high internal phase Pickering and traditional emulsions.

Figure 5c shows the effect of dilution of CaCO_3_ NPs-stabilized Pickering emulsions and TW80-stabilized traditional emulsions on creaming and droplet characteristics. When diluted 2- to 5-folds, the emulsions stabilized by CaCO_3_ NPs soon stratified after 12 h at RT. What’s more, the lower aqueous phase’s height was proportional to the dilution ratio used. In contrast, the TW80 control group’s diluted emulsions were not layered within 12 h because the stratification was too slow to render apparent phase separation. After 7-day storage, the diluted TW80 emulsions stratified dramatically. Despite the occurrence of stratification, dilution (2- to 5-folds) and storage time (7 d) had no apparent effects on the shapes of droplets and mean diameters (Fig. 5d–h). Hence, CaCO_3_ NPs-stabilized Pickering emulsions and TW80-stabilized traditional emulsions showed comparable and satisfying dilution stability.

### Encapsulation of VD3

**Fig. 6:**
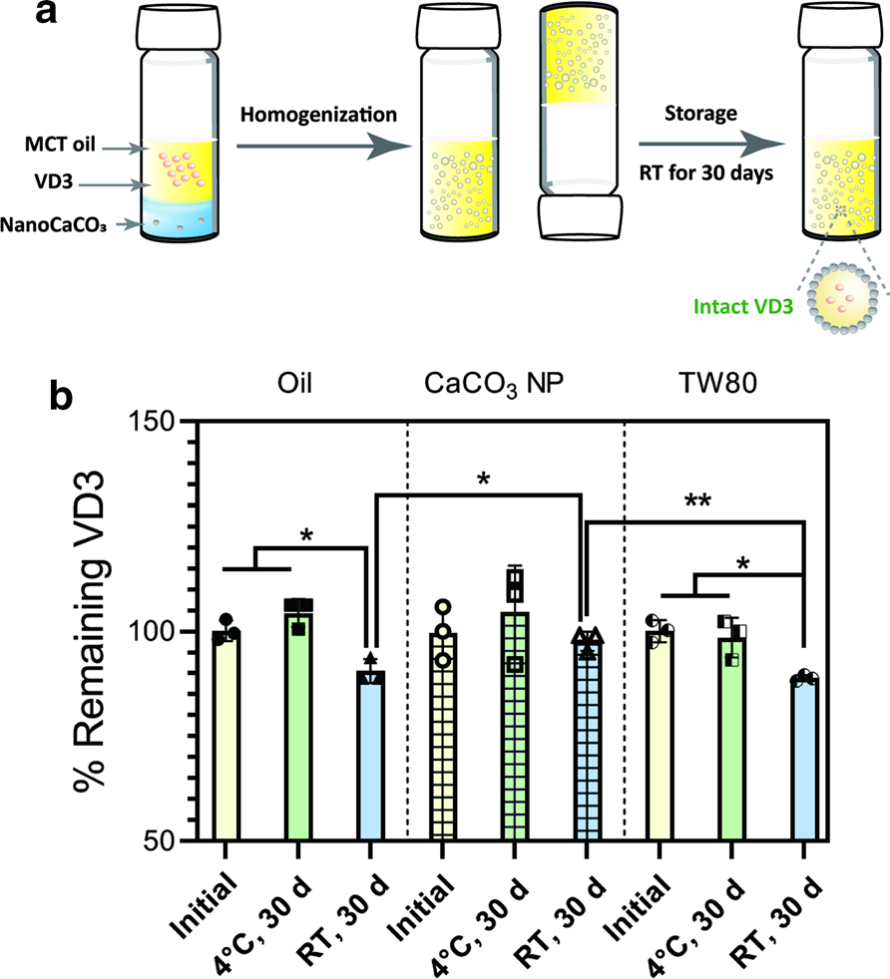
Enhanced storage stability of VD3 encapsulated in the CaCO_3_ NPs-based Pickering emulsion. **a** Schematic illustration showing the improved stability of VD3 after storage in the CaCO_3_ NPs emulsion compared to two controls, MCT and the emulsion stabilized by TW80. **b** Percentage of remaining VD3 after 30-day storage at RT and at 4 °C (*p < 0.05, and **p < 0.01). Data are mean ± standard deviation of the triplicates

HIPE shows great promise as an effective delivery system because it stabilizes a high volume of oily phase that is compatible with lipophilic substances. Encouraged by the desired stability of CaCO_3_ NP-based stabilized HIPE (*φ* = 0.8), we exploited it as a delivery vehicle for lipophilic compounds using VD3 as a model substance (Fig. 6a) and analyzed the remaining percentage of the encapsulated VD3 (Fig. 6b) at 4 °C and RT. For a fair comparison, VD3 was not only dissolved in MCT oil alone but also loaded in the TW80 stabilized emulsion at the same concentration, and then both these two VD3-containing samples were processed in the same manner as the CaCO_3_ NPs group. The results showed VD3 contents of the three groups did not decrease significantly after 30-day storage at 4 °C. However, when stored at RT for 30 days, the content of remaining VD3 was significantly reduced to 90.5 ± 2.8 % for MCT and 88.9 ± 0.7 % for the TW80 control, respectively, in contrast to no significant decrease in VD3 (*p* > 0.05) in the CaCO_3_ NPs group. It could be therefore calculated that about 10 % of VD3 loss occurred in the two control groups, which could be due to oxidative degradation [2]. For the CaCO_3_ NPs group, the remaining percentage of VD3 remained unchanged after storage at RT. An analysis of mean droplet size also confirmed the stability of the stored emulsions (Additional file 1: Figure S9). A comparison of the VD3 retention percentage revealed that the emulsified MCT oil environment (*φ* = 0.8) did not necessarily protect VD3 against degradation during the 30-day period. It is worth noting that the emulsion with a larger mean droplet size, in turn, results in a smaller specific surface area, and vice versa. Therefore, the high loss of VD3 in the TW80-stabilized emulsion may partially correspond to its higher specific surface area (385.1 ± 26.2 m^2^/kg of the TW80 group versus 95.9 ± 14.3 m^2^/kg of the CaCO_3_ NPs group). It has been proposed that viscosity could affect oxidation by reducing the diffusion of potential oxidizing substances [45]. Considering the dramatic difference in viscosity between the TW80- and CaCO_3_ NPs stabilized emulsions (Fig. 3), we assume that the higher viscosity of the CaCO_3_ NPs stabilized emulsion is partially responsible for the enhanced stability of VD3 through reducing the diffusion of oxidizing factors (e.g., oxygen).

### Responsiveness of the CaCO_3_ NP-based Pickering emulsion under simulated in vitro GI conditions

**Fig. 7:**
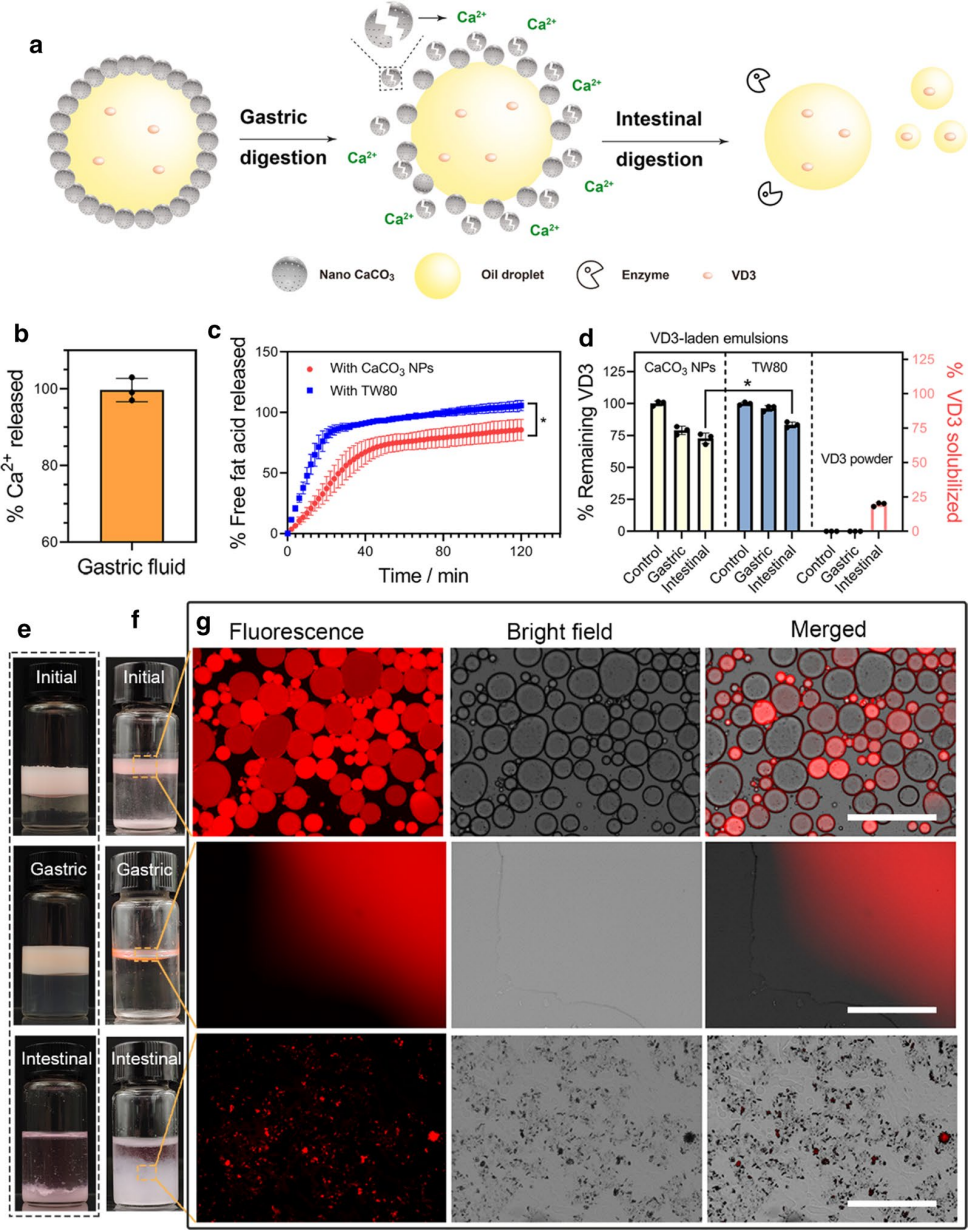
Gastrointestinal fate of the VD3 loaded CaCO_3_ NPs stabilized emulsion in simulation mode. **a** Schematic illustration of the digestion of CaCO_3_ NPs stabilized Pickering emulsion in simulated gastric and intestinal fluids. **b** Percentage of Ca^2+^ generated by gastric acid hydrolysis from CaCO_3_ NPs. **c** FFA released during the simulated intestinal lipolysis over time. Data points are mean ± standard deviation of the triplicates. An asterisk indicates statistically significant (*p* < 0.05) differences among samples. **d** Percentage of remaining VD3 after gastric and intestinal (left y-axis) and the percentage of VD3 solubilized in the simulated intestinal fluid after 2-h digestion (right y-axis). Data points are mean ± standard deviation of the triplicates. An asterisk indicates statistically significant (*p* < 0.05) differences among samples. Visual appearance of the diluted emulsion before and after simulated gastric and intestinal phases: **e** TW80, **f** CaCO_3_ NPs. **g** Fluorescent imaging of the oily phase of the emulsion and chyme obtained after simulated gastric and intestinal phases with CaCO_3_ NPs. The scale bar: 400 µm

For an edible Pickering emulsion formulation, it is necessary to understand the digestibility in the GI tract. Hence, we performed simulated in vitro gastric and intestinal digestions to highlight the fate of the VD3-loaded CaCO_3_ NPs stabilized Pickering emulsion (depicted by the schematic, as shown in Fig. 7a). Analysis of visual appearance was first carried out to show that the demulsification suffered in the simulated gastric fluid (pH 2.5). Upon digestion by acid, emulsion droplets stabilized by CaCO_3_ NPs rather than TW80 (Fig. 7e) coalesced into a bulk upper oil phase (Fig. 7f) because the CaCO_3_ NPs initially adsorbed onto the oil droplet surface were completely decomposed into Ca^2+^, as verified by the quantification analysis of Ca^2+^ (Fig. 7b). Similar demulsification by acid has been reported for emulsions stabilized by hydroxyapatite or calcium phosphate particles [11, 12]. From the standpoint of dietary calcium, such acid-responsive disintegration of the CaCO_3_ NPs would be an effective means for providing Ca^2+^ for physiological use. After gastric digestion at a neutral pH (7.4), the demulsified oil phase was depleted by the simulated intestinal fluid after 2-h digestion. The total percentage of FFA released was estimated to be 85.7 ± 8.7 % and 105.7 ± 4.1 % for the nano CaCO_3_- and TW80-stabilized emulsions, respectively, based on their cumulative releases of FFA (Fig. 7c). Considering the result obtained in this work, calcium ions that were liberated from the stabilizing CaCO_3_ NPs to the intestinal fluid may, on the one hand, decrease the FFA content because they may retard the micellization of oil during lipolysis and, on the other hand, form precipitates with free fatty acids [28]. When used as a delivery vehicle ingested orally, maintaining a satisfactory percentage of cargo remaining after the GI tract would be significant to predict the bioavailability of the cargo. For this reason, changes in the VD3 content were analyzed for the representative HIPE (*φ* = 0.8) when treated by gastric and intestinal digestion. The result showed that VD solubilized in the hydrolysate decreased to 79 ± 3.3 % and 72.8 ± 4.1 % for the CaCO_3_ NPs stabilized emulsion after stepwise gastric and intestinal digestions, respectively, and 96.2 ± 2.3 % and 83.3 ± 2.3 % for the TW80 stabilized emulsion (Fig. 7d). VD3 appeared to be better protected by the TW80 emulsion from degradation by gastric acid, which may be related to the stability in the gastric fluid (Fig. 7e). During the intestinal digestion, the oil phase of the TW80 emulsion was digested more thoroughly (Fig. 7c), which may promote the micellization of oil and VD3. In both cases, although the loss of one part of VD3 did take place, the final remaining percentage of VD3 was much higher with comparison to the control sample (VD3 powder) which presented a value of 19.6 ± 1.4 % (Fig. 7d). These quantitative results concerning Ca^2+^ and VD3 indicate that the VD3-containing HIPE may be useful as a calcium/VD3-fortified Pickering formulation.

To microscopically detail the demulsification process with CaCO_3_ NPs in simulated GI conditions (Fig. 7f g), we characterized the dispersed oil component using fluorescent microscopy. Initially, micrometered oil droplets bearing red fluorescence were clearly imaged. (Fig. 7f), which were digested into small fragments in the following simulated intestinal environment. This result indicated that the oil layer was partially broken into small-sized droplets during the intestinal digestion process [46].

### Oral toxicity assessment of CaCO_3_ NPs stabilized emulsion in vivo

**Fig. 8:**
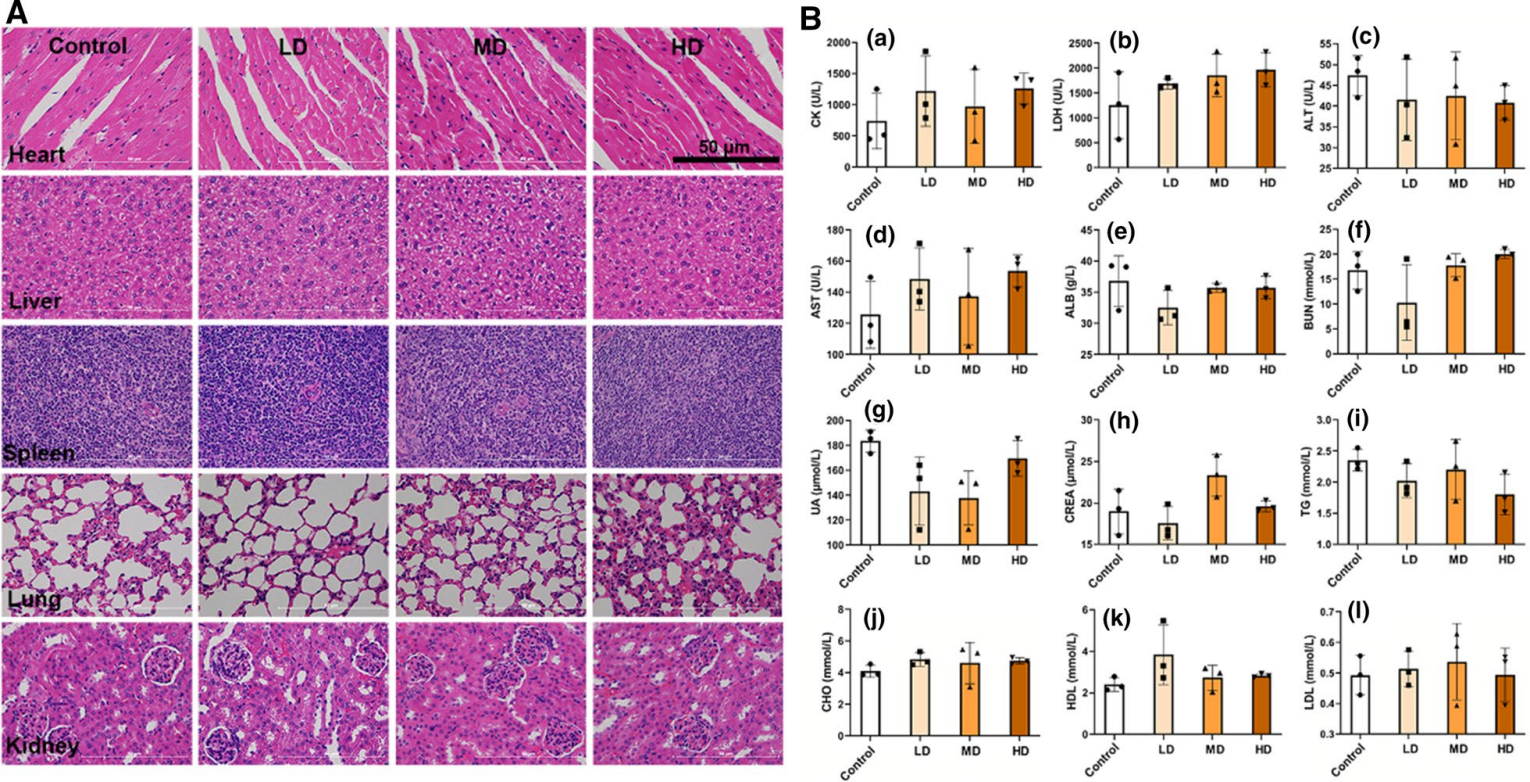
In vivo oral toxicity assessment. Oral toxicity of CaCO_3_ NPs stabilized emulsions to mice assessed by (**A**) H&E staining of heart, liver, lungs, spleen, and kidneys slices and (**B**) hematological analyses, including CK (**a**), LDH (**b**), ALT (**c**), AST (**d**), ALB (**e**), BUN (**f**), UA (**g**), CREA (**h**), TG (**i**), CHO (**j**), HDL (**k**), and LDL (**l**). Mice from the treated groups were gavaged with CaCO_3_ NPs stabilized emulsions containing 325 mg/kg (L), 650 mg/kg (M), and 1300 mg/kg (H) of CaCO_3_ NPs. Mice of the control received 0.9 % NaCl

As a carrier for VD3 for oral administration, the safety of the CaCO_3_ NPs stabilized MCT-water emulsion should be a primary concern. Hence, we conducted analyses of histopathology and blood biochemistry to study the in vivo toxicity due to the oral administration of CaCO_3_ NPs stabilized emulsion. The results showed no pathological difference in the heart, liver, spleen, lungs, and kidneys between the three treated and control groups (Fig. 8A). Also, the safety of the CaCO_3_ NPs stabilized emulsion was supported by the blood indices, including creatine kinase (CK), lactate dehydrogenase (LDH), alanine aminotransferase (ALT), aspartate aminotransferase (AST), albumin (ALB), blood urea nitrogen (BUN), uric acid (UA), and creatinine (CREA) (a–h in Fig. 8B). Interestingly, no difference in the four blood fat indicators (i.e., triglycerides: TG, cholesterol: CHO, high-density lipoprotein: HDL, and low-density lipoprotein: LDL, i–l in Fig. 8B) occurred between treated and control groups, indicating the intake of MCT during the administration of CaCO_3_ NPs stabilized emulsion caused no additional burden to blood fat. Although a more extended period is required to systematically evaluate the toxicity of the CaCO_3_ NPs stabilized emulsion, the promising results of the current short-term toxicity assessment (Fig. 8) suggest that this type of CaCO_3_ NPs stabilized emulsions is of low toxicity and has potential food, pharmaceutical, and biomedical applications.

## Conclusions

In summary, in this study, we prepared a type of bioresponsive CaCO_3_ NP-based Pickering emulsion with drug-delivering and calcium-fortifying properties. This emulsion design can overcome the drawbacks of a physical blend of CaCO_3_ NPs and VD3 and achieve the following advantages: (i) The stabilizing CaCO_3_ NPs robustly protect the emulsified droplet against coalescence, which enhances the stored stability of the encapsulated VD3. (ii) The surface-adsorbed CaCO_3_ NP layer results in the release of Ca^2+^ and gastric acid-trigged demulsification after exposure to simulated gastric fluid, which favors subsequent lipolysis in the following intestinal phase. (iii) The digestion of oil into FFA induces the release of VD3, which probably synergistically enhances the absorption rate of calcium. Together, this study not only provides a type of Pickering emulsion-based encapsulation and delivery system by using CaCO_3_ NPs as the emulsifier and MCT as the internal phase but also sheds light on the usage of the CaCO_3_ NP-based stabilized Pickering emulsions for an edible purpose, particularly as calcium-fortified Pickering emulsions.

## Supplementary Information

**Additional file 1: Figure S1.** Morphological change of the CaCO_3_ NPs after exposure to different pH conditions for 30 min. **Figure S2.** Mean droplet diameter, droplet size distribution, and micrographic of CaCO_3_ NP- and TW80-stabilized emulsions prepared under different shearing speeds. **Figure S3. **Insufficient emulsification performance of CCaCO_3_ and CaCO_3_ NPs at different concentrations. **Figure S4.** MCT/water emulsions fabricated with TW80 at different parameters. **Figure S5.** Calcium contents of the CaCO_3_ NPs and CCaCO_3_. **Figure S6.** Immiscibility of MCT and different oils and the separation of MCT and VD3 on a C18 column. **Figure S7.** Interfacial tension between the MCT/water interface decreased upon the addition of CaCO_3_ NPs or TW80. **Figure S8.** Shear viscosity of CaCO_3_ NPs dispersions at different concentrations. **Figure S9.** Changes in mean droplet diameter before and after storage at RT for 30 days.

## Data Availability

All data generated or analyzed during this study are included in this article.

## Abbreviations

GRAS: Generally regarded as safe
VD3: Vitamin D3
MCT: Medium-chain triglyceride
TW80: Tween 80
Cur-6: Cumarin-6
RB: Rhodamine B
GI: Gastrointestinal
PBS: Phosphate-buffer saline
XRD: X-ray diffractometer
XPS: X-ray photoelectron spectroscopy
FTIR: Fourier transform infrared spectra
TEM: Transmission electron microscopy
EDS: Energy-dispersive X-ray spectroscopy
SEM: Scanning electron microscopy
HPIC: High-performance ion-exchange chromatography
DSD: Droplet size distribution
RT: Room temperature
HPLC: High-performance liquid chromatography
UV: Ultraviolet
FFA: Free fatty acids
NIH: National institutes of health
H&E: Hematoxylin and eosin
ANOVA: One-way analysis of variance
HIPE: High internal phase emulsion
CK: Creatine kinase
LDH: Lactate dehydrogenase
ALT: Alanine aminotransferase
AST: Aspartate aminotransferase
ALB: Albumin
BUN: Blood urea nitrogen
UA: Uric acid
UREA: Urea
CREA: Creatinine
TG: Triglycerides
CHO: Cholesterol
HDL: High-density lipoprotein
LDL: Low-density lipoprotein
