# Supplementary material for: Edible CaCO3 nanoparticles stabilized Pickering emulsion as calcium‐fortified formulation
Source: J Nanobiotechnology. 2021 Mar 4;19:67. doi: 10.1186/s12951-021-00807-6 (PMC7934247; doi:10.1186/s12951-021-00807-6)
Supplement: Supplementary file 1 — Additional file 1: Figure S1. Morphological change of the CaCO3 NPs after exposure to different pH conditions for 30 min. Figure S2. Mean droplet diameter, droplet size distribution, and micrographic of CaCO3 NP- and TW80-stabilized emulsions prepared under different shearing speeds. Figure S3. Insufficient emulsification performance of CCaCO3 and CaCO3 NPs at different concentrations. Figure S4. MCT/water emulsions fabricated with TW80 at different parameters. Figure S5. Calcium contents of the CaCO3 NPs and CCaCO3. Figure S6. Immiscibility of MCT and different oils and the separation of MCT and VD3 on a C18 column. Figure S7. Interfacial tension between the MCT/water interface decreased upon the addition of CaCO3 NPs or TW80. Figure S8. Shear viscosity of CaCO3 NPs dispersions at different concentrations. Figure S9. Changes in mean droplet diameter before and after storage at RT for 30 days. [file 12951_2021_807_MOESM1_ESM.docx]

**Additional file**

**for**

**Edible CaCO_3_ nanoparticles stabilized Pickering emulsion as calcium-fortified formulation**

Xiaoming Guo^#^, Xiaoying Li^#^, Leung Chan, Wei Huang, Tianfeng Chen*

Department of Oncology, The First Affiliated Hospital, and Department of Chemistry, Jinan University, Guangzhou 510632, China

E-mail: tchentf@jnu.edu.cn.

Supplementary Results


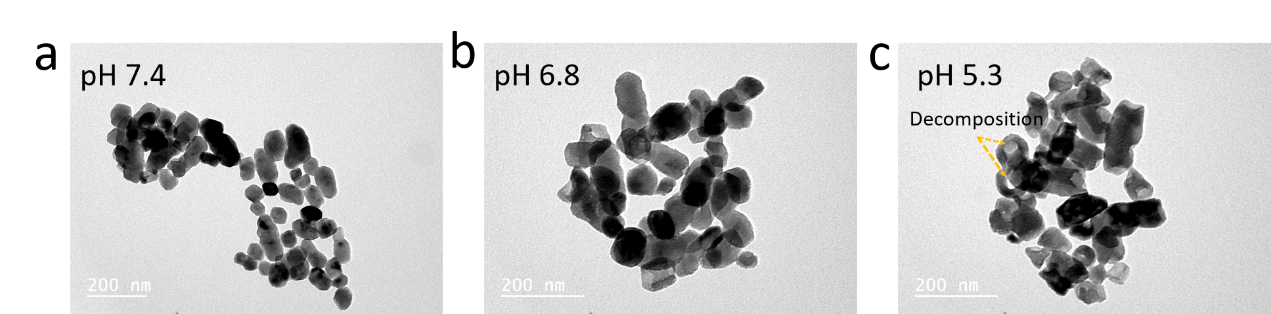


**Figure S1.** **Morphological change of the CaCO_3_ NPs after exposure to different pH conditions for 30 min.** (**a-b**) At pH 6.8 and 7.4, the CaCO_3_ NPs remained intact; however, at pH 5.3, the particle disintegration occurred (**c**).


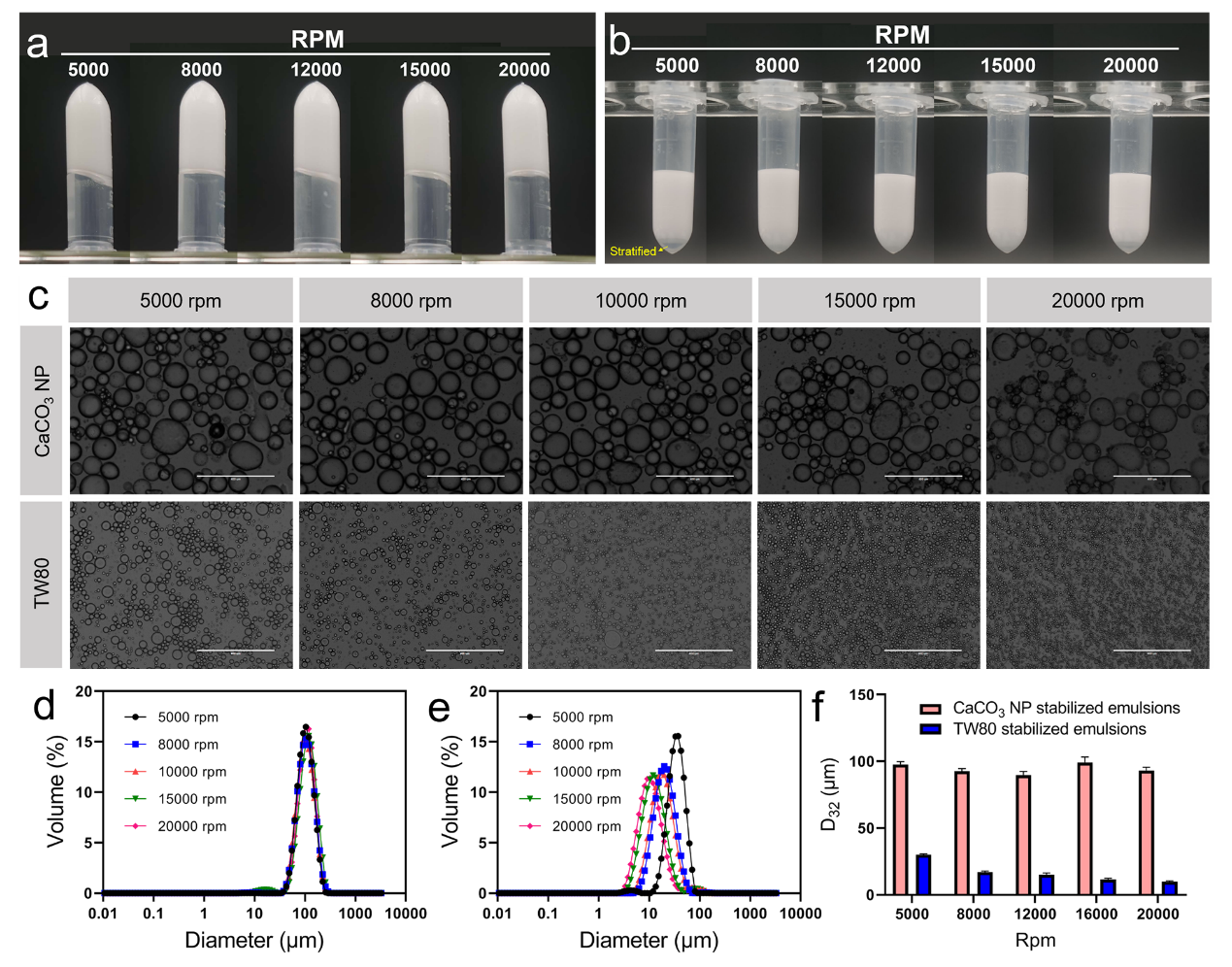


**Figure S2.** **Mean droplet diameter, droplet size distribution, and micrographic of CaCO_3_ NP- and TW80-stabilized emulsions prepared under different shearing speeds.** Photographs of MCT-water emulsions (8:2) stabilized by 1% w/v CaCO_3_ NP (**a**) and 0.5% w/v TW80 (**b**) taken 24 h after preparation. Each emulsion was prepared by first mechanically shearing the immiscible MCT-water mixture at different shearing speeds (5,000–20,000 rpm) for 2 min. In panels a and b, shearing speeds from left to right are 5,000, 8,000, 12,000, 15,000, and 20,000 rpm. (**c**) Micrographs of MCT-in-water emulsions stabilized by 1% w/v CaCO_3_ NP and 0.5% w/v TW80 at different shearing speeds. Scale bars are 400 μm. Before microscopic observation, each emulsion was diluted 2 times with ultrapure water. (**d-e**) Droplet size distributions and (**f**) mean diameter sizes of MCT-water emulsions (8:2) stabilized by 1% w/v CaCO_3_ NP and 0.5% w/v TW80 at different shearing speeds. D_32_ data are mean ± standard deviation in duplicate.


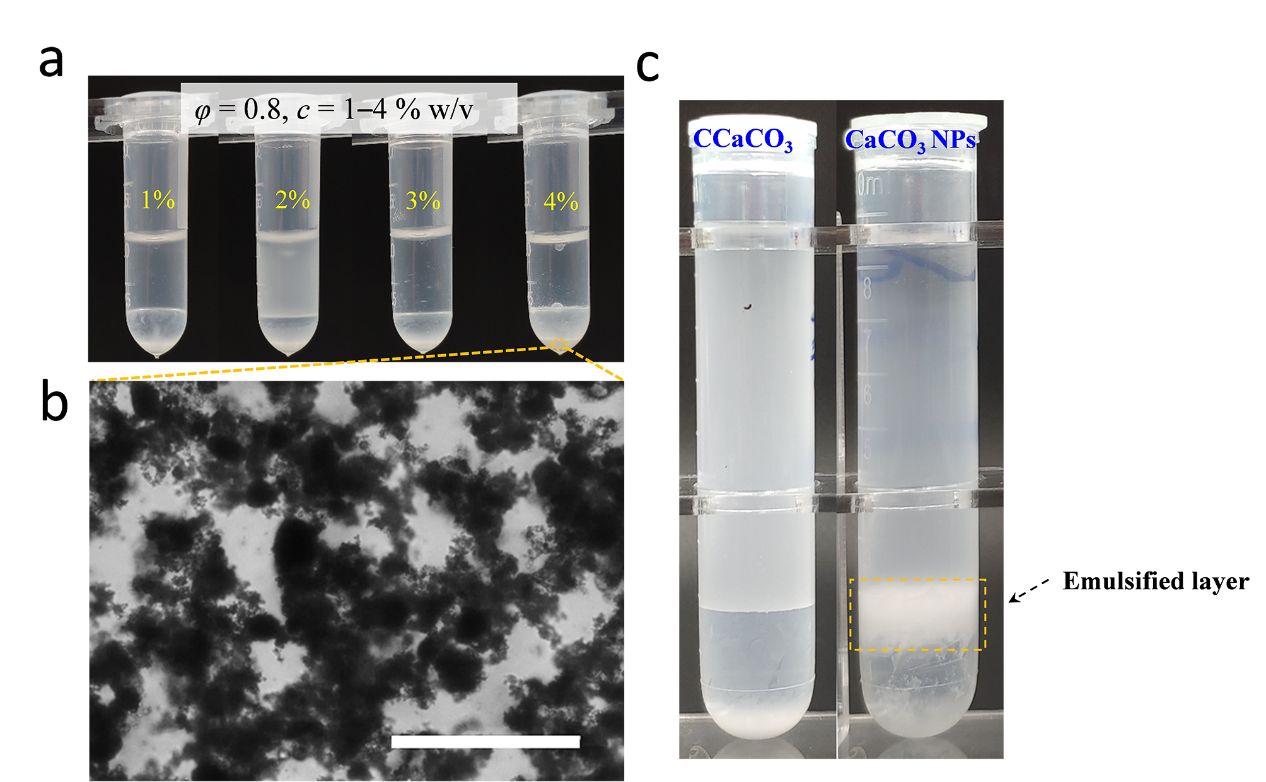


**Figure S3.** **Insufficient emulsification performance of CCaCO_3_ and CaCO_3_ NPs at different concentrations.** (**a**) Visual appearance of the two-phase mixtures containing MCT and the CCaCO3 suspension as the upper and lower phases, respectively. Samples were placed into vials just after preparation and then photos were taken after standing at room temperature for 12 h. (**b**) Micrographic image of the CCaCO_3_ precipitated at the bottom of the tube. The scale bar: 200 μm. (**c**) Digital photographs of the CCaCO_3_ and CaCO_3_ NPs stabilized oil-in-water emulsions taken 12 h after preparation. The *c* was 0.5 % (w/v) and *φ* was 0.8. The aqueous phase was prepared with PBS (20 mmol·L^-1^, pH 7.4).


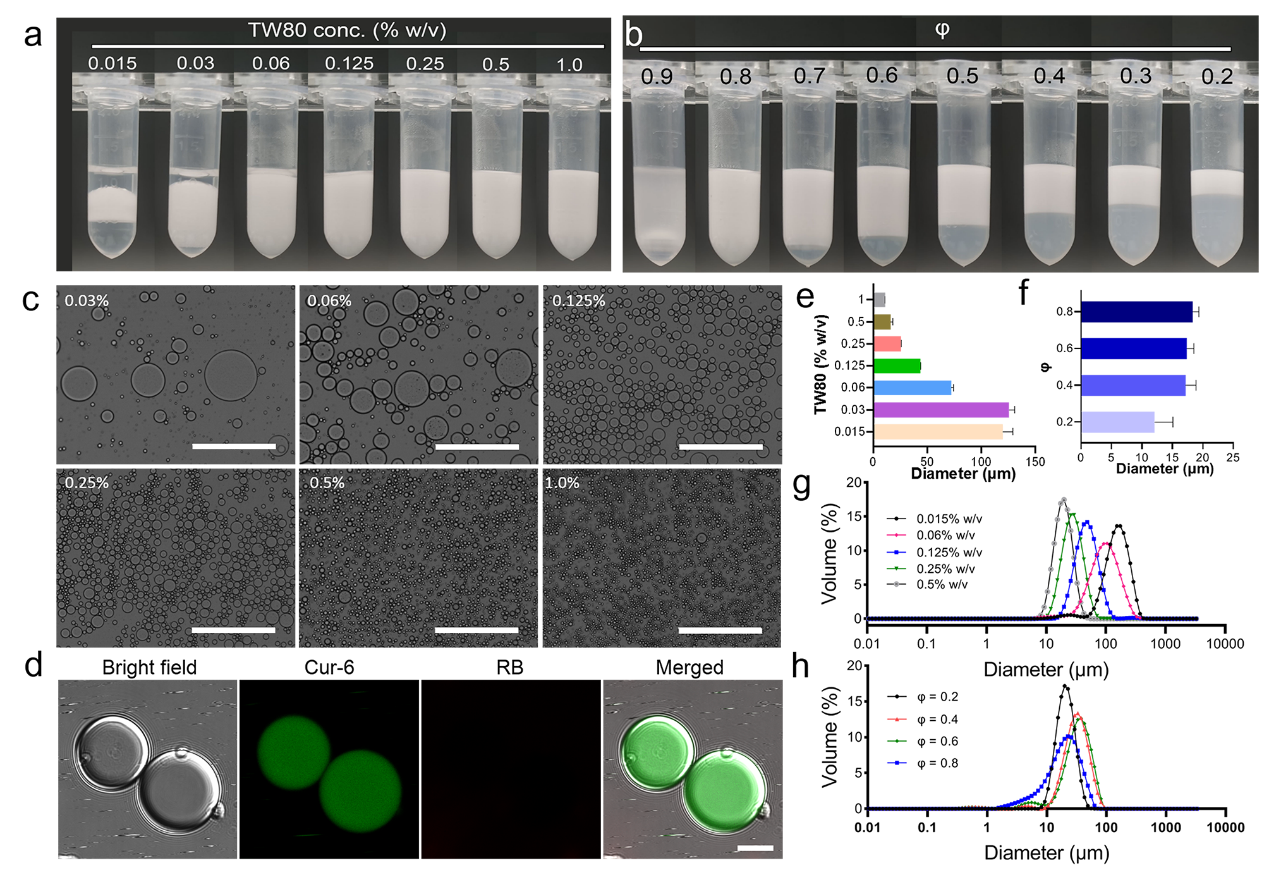


**Figure S4.** **MCT/water emulsions fabricated with TW80 at different parameters.** (**a**) Photographs of MCT-water emulsions (8:2) stabilized by TW80 at different concentrations (0.015–1.0% w/v). (**b**) Photographs of MCT-water emulsions stabilized by 0.5% w/v TW80 at different *φ* values ranging from 0.2 to 0.9. (**c**) Micrographs of selected MCT-water emulsions stabilized by TW80 at different concentrations. *φ* is 0.8, and the shearing speed is 8000 rpm. Scale bars indicate 400 μm. (**d**) Confocal laser scanning microscope images of droplets stabilized by 0.5% w/v TW80. Oil droplets with green fluorescences were dyed by Cur-6. The scale bar indicates 10 μm. (**e**) Mean diameter sizes and (**g**) droplet size distributions versus emulsifier concentration (0.015–1.0% w/v) for TW80 stabilized MCT-water emulsions at *φ* = 0.8. (**f**) Mean diameter sizes and (**h**) droplet size distributions versus *φ* (0.2–0.8) for MCT-water emulsions stabilized by 0.5% w/v TW80. D_32_ data are mean ± standard deviation in triplicate.


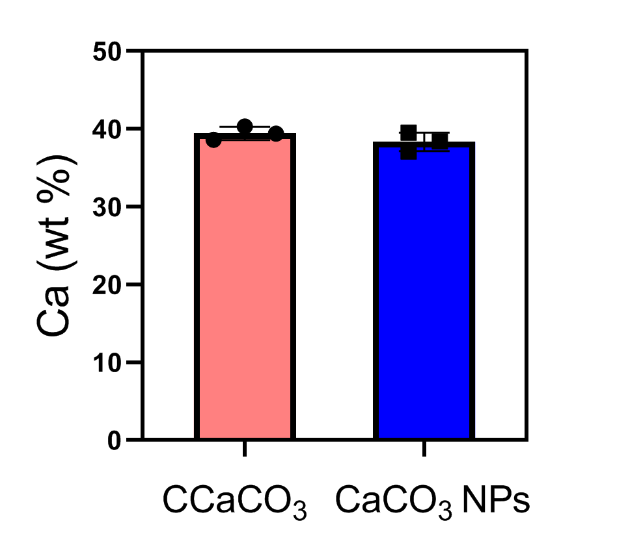


**Figure S5.** **Calcium contents of the CaCO_3_ NPs and CCaCO_3_.** Quantification of the calcium content was carried out using high-performance ion-exchange chromatography (HPIC). An adequate amount of sample was digested using concentrated methanesulfonic acid for 1 h and then diluted with a 20 mmol·L^-1^ methanesulfonic acid to set the calcium concentration into a linear range of 1–100 μg·mL^-1^. The determination of calcium content was performed as our previous work [1]. A calibration curve (linearity = 0.999) was constructed using calcium chloride (99.99%, Macklin, China, C805228) as standard.

**
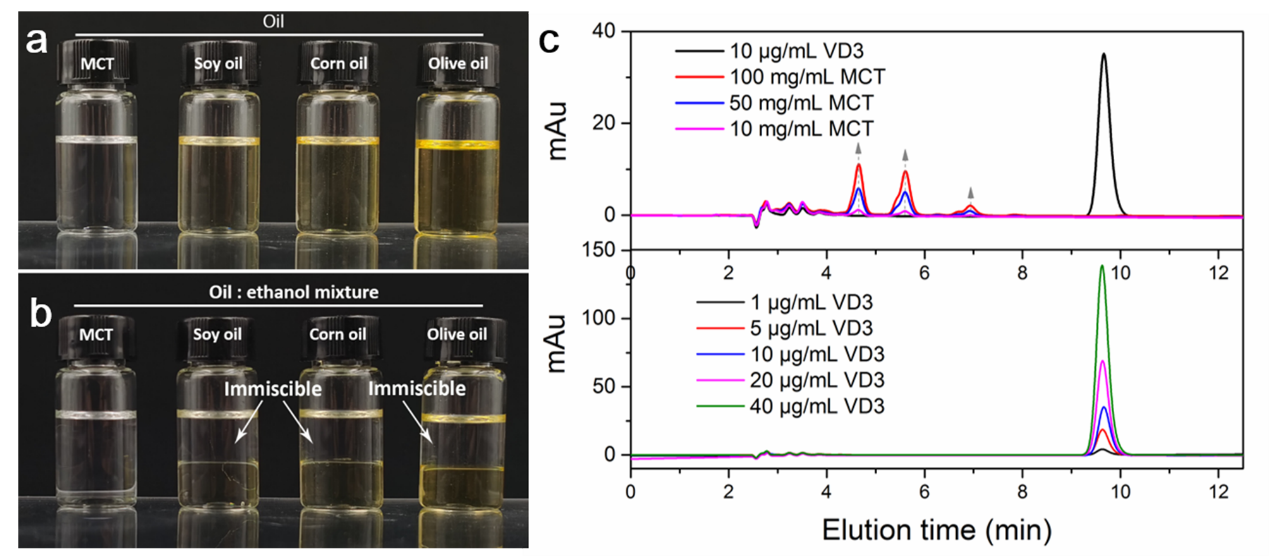
**

**Figure S6. Immiscibility of MCT and different oils and the separation of MCT and VD3 on a C18 column.** (**a**) Photographs of MCT, soy, corn, and olive oils solely and **(b)** their mixtures with an equal volume of ethanol taken 1 h after preparation. **(c)** Elution profiles of MCT and VD3 were recorded at 265 nm. The content of MCT and VD3 varies from 10-100 mg/mL and 1-40 ppm, respectively.


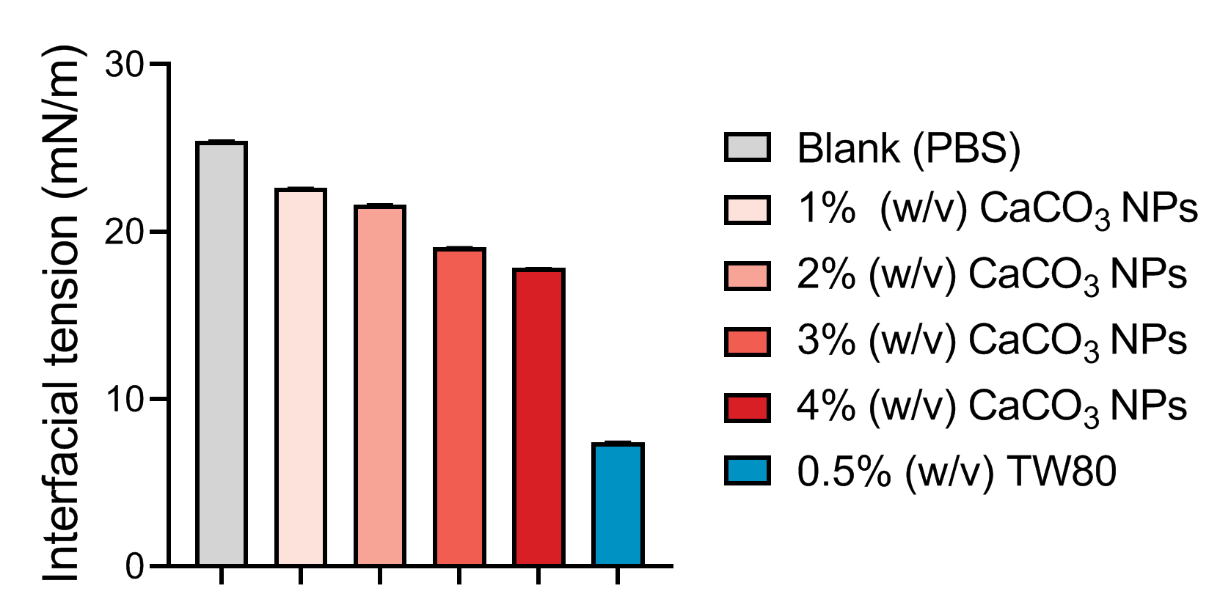


**Figure S7. Interfacial tension between the MCT/water interface decreased upon the addition of CaCO_3_ NPs or TW80.** Data are mean ± standard deviation in triplicate. A PBS solution (20 mmol·L^-1^, pH 7.4) was used as the blank. The interfacial tension was measured using a DCAT 21 interfacial tension-meter (Dataphysics Instruments Ltd., Germany) equipped with a platinum plate.





**Figure S8.** **Shear viscosity of CaCO_3_ NPs dispersions at different concentrations.** Data are mean ± standard deviation in duplicate. The steady shear viscosity η was measured at a shear rate γ̇ of 10 s^−1^. Measurements were conducted at 25 °C.


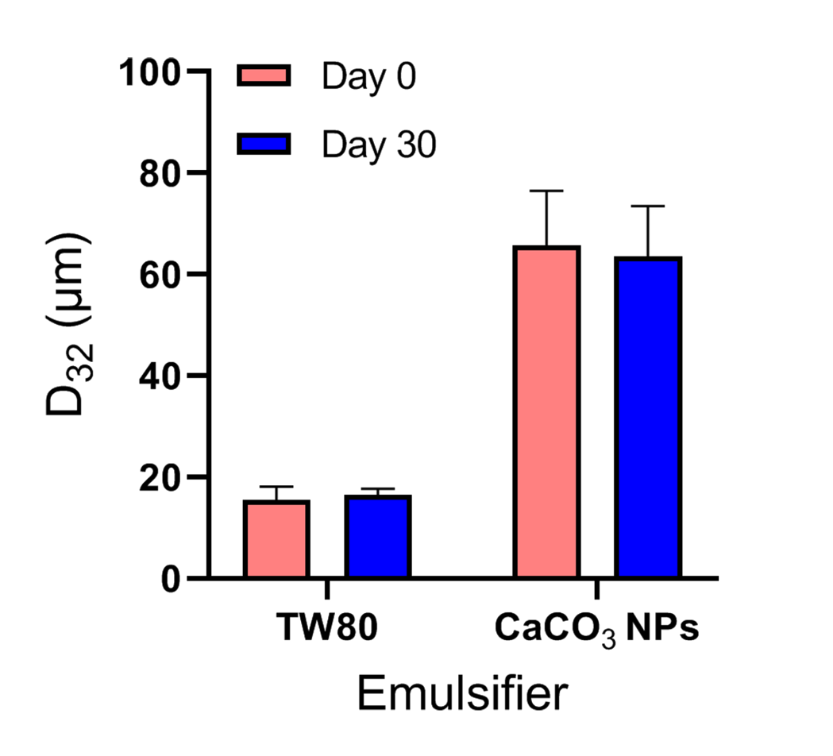


**Figure S9.** **Changes in mean droplet diameter before and after storage at RT for 30 days.** Mean droplet diameter of emulsions (*φ* = 0.8) stabilized by 4% w/v CaCO_3_ NPs or 0.5% w/v TW80, respectively. D_32_ data are mean ± standard deviation in triplicate. No significant difference (*p* > 0.05) in d_32_ was observed for each of the two groups after storage.

**Reference**

1. Guo X, Meng H, Tang Q, Pan R, Zhu S, Yu S: Effects of the precipitation pH on the ethanolic precipitation of sugar beet pectins**.** *Food Hydrocoll.* 2016, 52**:**431-437.
